# Supplementary material for: The prevalence of urogenital and intestinal schistosomiasis among school age children (6–13 years) in the Okavango Delta in Botswana
Source: PLoS One. 2023 May 30;18(5):e0285977. doi: 10.1371/journal.pone.0285977 (PMC10228809; doi:10.1371/journal.pone.0285977)
Supplement: Attachment — Submitted filename: Response to Reviewers _PONE-D-20-35623 July 2021.docx [file pone.0285977.s002.docx]

University of Botswana School of Nursing

Corner of Notwane and Mobuto Road

Private Bag 0022

Gaborone, Botswana

Date: July 12, 2021

**To:**	Academic Editor

PLOS One

Attention: Jazmin Toth

Dear Academic Editor

We are pleased to resubmit our manuscript PONE-D-20-35623 **Then prevalence of schistomiasis among primary school going children (6-13 years)in the Okavango Delta in Botswana** by Nthabiseng A. Phaladze (Principal investigator), and co-authors Lebotse Molefi, Olekae T. Thakadu, Onalenna Tsima, Barbara N. Ngwenya, Tuduetso L. Molefi and Wananani B. Tshiamo for consideration to be published in PLOS One.

We wish to take this opportunity once again to heartily thank the reviewers for their valuable time and useful contribution to this work. We appreciate the thorough inputs they have given which will no doubt help improve our manuscript.

The following paragraphs contain point-by-point responses to the reviewers’ and editorial board comments:

**Response to Comments by the Academic Editor:**

1. Reference to Table 5: reference is made to Table 5 on page 11 of the manuscript.
2. Affiliation of Mr. Lebotse Molefi: At the time this study was conducted, Mr. Molefi was an employee of the University of Botswana in the School of Allied Health Professions. His initial affiliation has been maintained. His current address is Quality Anchor Consultants PTY.LTD, Plot 1159, Bokaa. Botswana.
3. Mr. Lebotse Molefi’s affiliation with Quality Anchor Consultants PTY. LTD has nothing to do with this study and does not alter our adherence to PLOS ONE policies on sharing data and materials.
4. Mr. Lebotse Molefi’s affiliation has been modified in the title page and will be modified in the system as well. We further confirm that there are no patents, products in development or marketed products associated with this research to declare.
5. Affiliations of authors have been updated to reflect those held by the authors at the time the study was conducted.

We hope these responses meet your expectations and awaiting your decision.

Sincerely,

Nthabiseng A. Phaladze PhD.

Professor of Nursing

University of Botswana School of Nursing

Email: phaladze@ub.ac.bw

Alternate email: nphaladze@yahoo.co.uk
