# Supplementary material for: The prevalence of urogenital and intestinal schistosomiasis among school age children (6–13 years) in the Okavango Delta in Botswana
Source: PLoS One. 2023 May 30;18(5):e0285977. doi: 10.1371/journal.pone.0285977 (PMC10228809; doi:10.1371/journal.pone.0285977)
Supplement: Attachment — Submitted filename: Response to Reviewers_PONE-D-22-26018 JANUARY 20 2023.docx [file pone.0285977.s003.docx]

P.O.Box 70229

Gaborone

Botswana

Date: January 20, 2023

**To:**	Academic Editor

PLOS One

**Attention:** Dr. Clement Ameh Yaro

Dear Academic Editor

We are pleased to resubmit a manuscript PONE-D-22-26018 **The prevalence of urogenital and intestinal s*chistosomiasis* among school age children (6-13 years) in the Okavango Delta in Botswana** by Nthabiseng A. Phaladze (Principal investigator), and co-authors Lebotse Molefi, Olekae T. Thakadu, Onalenna Tsima, Barbara N. Ngwenya, Tuduetso L. Molefi and Wananani B. Tshiamo for consideration to be published in PLOS One.

We wish to take this opportunity to heartily thank the reviewers for their valuable time and useful contribution to this work. We appreciate the thorough inputs they have given which will no doubt help improve our manuscript.

The following paragraphs contain point-by-point responses to the reviewers’ and editorial board comments:

A. **Response on Journal Requirements:**
1. **Compliance of the Manuscript to PLOS ONE’s style requirements:** We are happy to submit the revised manuscript in line with all the requirements of the journal.
2. Competing Interests Statement: I declare that the authors have no conflict of interest. Mr . Lebotse Molefi is a paid employee of Quality Anchor Consultants PTY. LTD, but was not affiliated with the study at the time of this study was conducted. There are no patents, products in development or marketed products associated with this research to declare. This does not alter our adherence to PLOS ONE policies on sharing data and materials.”
3. **Funder:** Funding information has been removed from the Acknowledgments section. We would like to maintain the Funding Statement as it reads "This study was funded by the National Institute of Health Research (Grant Number: 16/136/33 NIHR). The funders had no role in study design, data collection and analysis, decision to publish, or preparation of the manuscript.” Any additional information provided contrary to the original statement should be deleted.
4. **Copyright of Figures 1 & 2:** **have been deleted** as advised since it was difficult to get permission from the copyright holder.
   B. **Comments to the Author:**
      1. Is the manuscript technically sound, and do the data support the conclusions?
         We note that Reviewers agree on this aspect. We have also noted comments made by Reviewer #1.
      2. Has the statistical analysis been performed appropriately and rigorously?

We note that Reviewers agree on the statistical analysis.

3. Have the authors made all data underlying the findings in their manuscript fully available?

   The [PLOS Data policy](http://www.plosone.org/static/policies.action#sharing) requires authors to make all data underlying the findings described in their manuscript fully available without restriction, with rare exception (please refer to the Data Availability Statement in the manuscript PDF file). The data should be provided as part of the manuscript or its supporting information or deposited to a public repository. For example, in addition to summary statistics, the data points behind means, medians and variance measures should be available. If there are restrictions on publicly sharing data—e.g. participant privacy or use of data from a third party—those must be specified.

We also note that Reviewers agree on this aspect.

4. Is the manuscript presented in an intelligible fashion and written in standard English?

PLOS ONE does not copyedit accepted manuscripts, so the language in

submitted articles must be clear, correct, and unambiguous. Any

typographical or grammatical errors should be corrected at revision, so please

note any specific errors here.

We do note the Reviewers divergent views on this question; but do take comments made by Reviewer #1 seriously and have attended to typographical and grammatical errors.

5. **Review Comments to the Author:**
   a. ***Reviewer #1***

1. **Title:** The suggestion to change the title to “The prevalence of urogenital and intestinal s*chistosomiasis* among school age children (6-13 years) in the Okavango Delta in Botswana” has been adopted.

The phrase “primary school aged children (6-13 years)” in the title and throughout in the document has been replaced with “school-age children in the title and throughout the text.

2. **Abstract:** Page 1, Background: line 1, the study purpose has been provided “This study sought to investigate prevalence and intensity of schistosomiasis among school age children 6-13 years in selected communities in the Okavango Delta”.

- Methods: More details of methods used for stool and urine examination, as well as for statistical analysis of data have been provided.

- Recommendation preceded by main findings as per advice.

3. Page 7, Specific objectives have been removed, and the study purpose maintained.
4. Page 7, Heading created “Study area and population”.
5. Ethical considerations: statement on assent by children corrected on page 9, lines 156-157.
6. The statement that reads “Total Urine (n=1603) and stool (n=1404) samples collected from school aged children from all the selected schools to determine the prevalence and intensity of schistosomiasis.” was re-phrased as suggested to read “A total of 1603 urine and 1404 stool samples were collected from school age children in the selected schools to determine the prevalence and intensity of schistosomiasis.” More details on methods used to analyze urine and stool samples are provided on Page 10.
7. Comment: For examination of S. haematobium infection, WHO recommends urine filtration method. It is not clear why the authors used additional centrifugation. The authors need to clarify this.

- **Clarification:** The majority of intestinal, urinary and blood parasites

can be detected microscopically in unstained or stained preparations,

either directly or following concentration by centrifugation. (Medical

Laboratory Manual for Tropical Countries Volume 1 page 178 (second

edition by Monica Cheesbrough (ELBS-English Language Book

Society/Tropical Health Technology/ 1987).

To increase the sensitivity of detecting parasite ova, urine filtration

and centrifugation methods were used to analyze urine samples

concurrently. Positive diagnosis of *S. haematobium* was based on the

detection of one terminal spined schistosoma ovum or more.

8. The urine and stool examination methods have been re-written and appropriate references cited.
9. Comments on Tables 2;5;6;7 &8 have been addressed and statistical corrections done. The issue of other soil transmitted helminths -Ascaris, Taenia have been removed from the tables. PLOS ONE does not allow footnotes. We added a sentence in the text to acknowledge that even though other STHs were not the focus, during urine and stool examination, they were identified.
10. The statement “Our data provide baseline information regarding infection status of Schistosoma and STH among school aged children living along the Okavango Delta.” The statement has been replaced with a summary of the findings.
    b. ***Reviewer #2:***
       1. Integer inserted in all Figures given in all the tables and throughout the

manuscript.

2. Line 66: Referencing of Hajissa et al., 2018 corrected.
3. Lines 85; 87; 183 & 187 corrected as per advice.
4. Methods of examination for urine and stool samples written in a

narrative form; bullets removed.

5. Lines 349, 359 and 412: Genus and species names corrected as per

advice.

6. Repetition of information removed, and grammar corrected.
7. Lines 433 and 436: Reconstruction of the sentence corrected as

suggested.

8. Line 461: Covid-19 restriction accepted.
9. References Section corrected.

We hope these responses meet your expectations and awaiting your decision.

Sincerely,

Nthabiseng A. Phaladze PhD.

Professor of Nursing

University of Botswana School of Nursing

Email: phaladze@ub.ac.bw

Alternate email: nphaladze@yahoo.co.uk
